# QCL-Based Dual-Comb Spectrometer for Multi-Species Measurements at High Temperatures and High Pressures

**DOI:** 10.3390/s20123602

**Published:** 2020-06-26

**Authors:** Guangle Zhang, Raphael Horvath, Dapeng Liu, Markus Geiser, Aamir Farooq

**Affiliations:** 1 Clean Combustion Research Center, Physical Sciences and Engineering Division, King Abdullah University of Science and Technology, Thuwal 23955, Saudi Arabia; guangle.zhang@kaust.edu.sa (G.Z.); dapeng.liu@kaust.edu.sa (D.L.); 2 IRsweep AG, Laubisruetistr. 44, 8712 Staefa, Switzerland; raphael.horvath@irsweep.com (R.H.) markus.geiser@irsweep.com (M.G.)

**Keywords:** dual-frequency combs, mid-infrared absorption spectroscopy, chemical kinetics, shock tube

## Abstract

Rapid multi-species sensing is an overarching goal in time-resolved studies of chemical kinetics. Most current laser sources cannot achieve this goal due to their narrow spectral coverage and/or slow wavelength scanning. In this work, a novel mid-IR dual-comb spectrometer is utilized for chemical kinetic investigations. The spectrometer is based on two quantum cascade laser frequency combs and provides rapid (4 µs) measurements over a wide spectral range (~1175–1235 cm^−1^). Here, the spectrometer was applied to make time-resolved absorption measurements of methane, acetone, propene, and propyne at high temperatures (>1000 K) and high pressures (>5 bar) in a shock tube. Such a spectrometer will be of high value in chemical kinetic studies of future fuels.

## 1. Introduction

For time-resolved chemical kinetic studies, a laser source with a wide spectral coverage and a fast tuning speed is highly desirable to achieve multi-species absorption measurements at rapid rates. Mid-infrared laser sources are being increasingly utilized as the vast majority of molecules have *fingerprint* spectral features in the mid-infrared region with absorption strengths orders of magnitude higher than in the near-infrared [1]. Some popular laser sources in mid-infrared are introduced in detail in [2], and include lead–salt diode lasers, sources based on optical parametric generation in nonlinear crystals, and quantum cascade lasers (QCLs). Among these, lead–salt lasers require cryogenic cooling, and are thus not quite user friendly, while sources based on nonlinear optics remain relatively complex with limited overall efficiency [3,4,5]. In contrast, QCLs are more compact and robust with a relatively narrow linewidth, and have thus attained popularity in various applications [6,7,8,9,10,11]. QCLs are unipolar semiconductor lasers which emit *via* intersubband transitions, with multiple quantum well heterostructures serving as the active region. Different types of mid-infrared QCLs, such as distributed feedback (DFB) QCLs working in continuous wave or pulse modes and external cavity QCLs, are introduced in detail in the literature [12,13]. Many chemical kinetic studies have been performed by using these QCLs [14,15,16,17,18,19,20,21]. Despite their success in providing time-resolved speciation measurements, these QCL can only provide narrow spectral coverage (~1–2 wavenumbers) in fast (~μs) measurements, and, therefore, these have mostly been applied for single species detection.

In this work, we employ an emerging spectroscopic tool, a QCL-based dual-comb spectrometer, which can enable multi-species measurements at rapid rates. The spectrometer is based on dual-comb spectroscopy which is proposed as an intriguing form of Fourier Transform spectroscopy technique with a dramatically increased temporal resolution. The working principles and important features of dual-comb spectroscopy technique are briefly described in Section 2.1. One may refer to [22] to gain a comprehensive knowledge of this interesting technique. Dual-comb spectroscopy could surpass conventional broadband spectroscopy for a wide range of applications as frequency comb technology progresses. There are other references [23,24,25,26,27,28,29,30,31,32,33,34,35,36,37,38,39,40,41,42,43,44,45,46,47] introducing different dual-comb spectroscopy techniques and applications in different fields, such as atmosphere monitoring, combustion, and biomedical studies. Here, we focus on dual-comb spectroscopy based on quantum cascade laser frequency combs (QCL-FC) due to their wide spectral coverage, high spectral resolution, fast time response, and a compact construction. QCL-FCs and their applications are discussed in the recent literature [40,48,49,50,51,52,53,54,55] and their operation / functionality is contrasted with other types of frequency combs in these references [44,56,57,58,59,60,61,62,63].

The dual-comb spectrometer presented in this work utilizes two commercial, stand-alone, all-solid-state, compact QCL FCs manufactured by Alpes Lasers [64,65] as the laser sources. The spectrometer enables microsecond-resolved broadband measurements of biological processes [36] or chemical kinetic processes [47], and shows great potential for surpassing Fourier Transform spectrometers [1] or rapid-tuning external cavity quantum cascade lasers [66] in terms of temporal resolution coupled with wide spectral coverage. In this work, we utilize this QCL-based dual-comb spectrometer to demonstrate rapid absorption measurements of methane (CH_4_), propene (C_3_H_6_), propyne (C_3_H_4_) and acetone (C_3_H_6_O) behind reflected shock waves at high temperature (> 1000 K) and high pressure (> 5 bar) in a shock tube to show its potential for rapid, multi-species measurements in chemical kinetic studies.

## 2. Materials and Methods

### 2.1. Dual-Comb Spectroscopy

**Figure 1:**
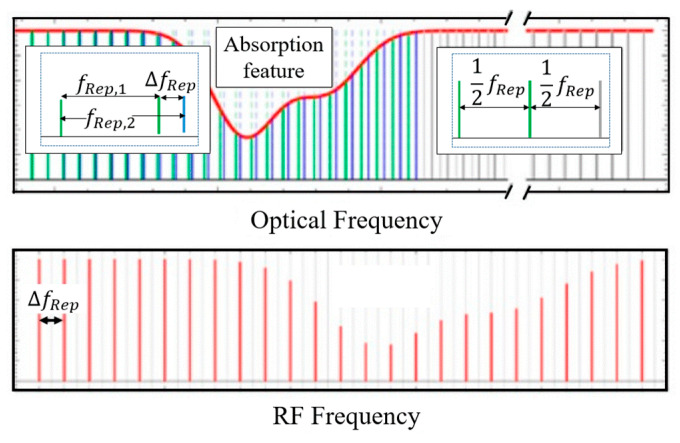
Top: Two frequency combs with slightly different repetition frequencies (f_Rep,1_, f_Rep,2_). Bottom: The beating signal of the interleaving combs showing how the information from the optical range is mapped on to the radio frequency range. Adapted from [36].

A dual-comb broadband spectrometer based on dual-comb spectroscopy (DCS) is used in this work for mid-IR absorption measurements. The generalized DCS concept is summarized and illustrated in Figure 1. Two optical frequency combs with slightly different repetition rates, f_Rep,1_ and f_Rep,2_, interfere on a single-pixel infrared detector, thereby generating a radio frequency (RF) comb spectrum, with a fixed spacing of Δf_Rep_ = f_Rep,2_ – f_Rep,1_, also called a multi-heterodyne spectrum. To perform spectroscopy, either one or both combs are passed through the desired sample. The absorption feature produces an attenuation in the comb intensity in the optical domain, and the attenuation induces a change in the RF beat signal. A change in the beatnote signal can be decoded to get attenuation and then absorption at each optical frequency. The minimum acquisition time, limited by T_res_ = 1/Δf_Rep_, can reach sub-microsecond. The frequency resolution (or instrument line shape) of the spectrometer is determined by the observed RF comb tooth linewidth over the measurement time. For a QCL frequency comb, the typical values of frequency resolution and comb spacing are <10^−4^ and ~0.3 cm^−1^, respectively. A dual-comb spectrometer usually has a trade-off between point spacing and acquisition time. 

### 2.2. Mid-IR Dual-Comb Spectrometer 

The table-top dual-comb spectrometer (IRis-F1, IRsweep) used in this work is based on two broadband QCL-FCs with an individual spectral coverage of > 70 cm^−1^, centered at ~1205 cm^−1^. By adjusting the temperatures and currents of the two combs, the spectrometer enables spectral coverage of about 60 cm^−1^ from the 1175 to 1235 cm^−1^ region with spectral sampling of 0.328 cm^−1^ and Δf_Rep_ of ~3 MHz. The two frequency-comb beams are combined using a 50:50 CaF_2_ beam splitter and attenuated with neutral density filters. One arm of the beam is focused directly on a high-bandwidth (1 GHz), AC-coupled HgCdTe (MCT) reference detector (PV-3TE-10.6, Vigo), while the other beam is transmitted through the test sample (e.g., a static cell or a shock tube) and focuses on a second identical MCT detector after passing through an ND filter and an optical immersion lens. The beating signal recorded by the detector is digitized and then analyzed using MATLAB. The pre-trigger intensity of the first 6 ms of each acquisition is averaged and taken as background signal. The post-trigger intensities are normalized by this background to form the time-resolved difference spectra which are the final output from the spectrometer. The absolute absorbance is then obtained from the difference spectra during post-processing. The total laser power at the sample is attenuated to ~1 mW to remain in the linear regime of the detector [36]. The spectral noise is line-dependent and correlates with light intensity at each line [67]. A typical RMS noise value of a strong line is 4 mOD at a time resolution of 4 μs, which scales to 400 μOD at a 1 ms time resolution [36]. The standard deviation of each transmission line is discussed in Section 3.1.

**Figure A1:**
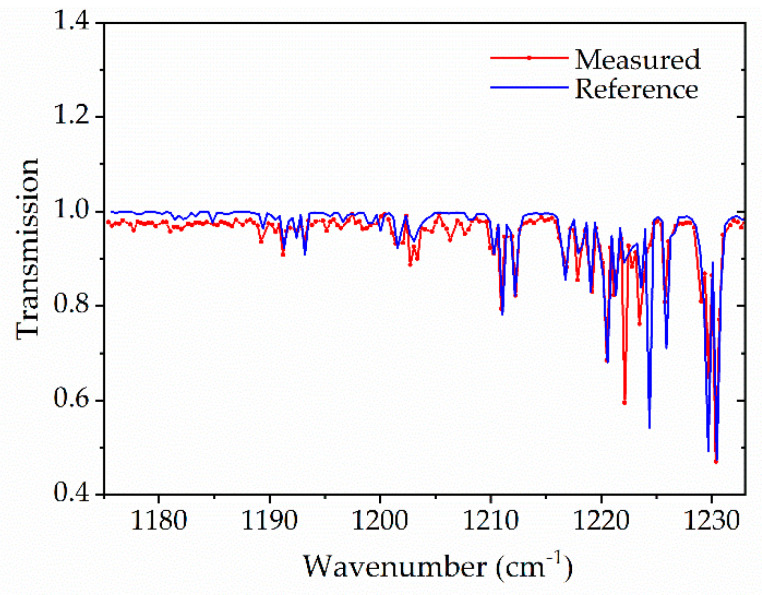
Measured and reference (HITRAN database) transmission spectrum of 5% methane/nitrogen at a temperature of 296 K and a pressure of 1.5 atm for wavelength calibration. Measured spectra were obtained in static conditions by the QCL-based dual-comb spectrometer.

Typically, the frequency axis of the spectrometer is calibrated with the help of a thin sample of solid polypropylene, which is very convenient to use. One would get the transmission spectrum by placing the polypropylene film in the laser path when there is no other test sample. In this work, we instead used 5% methane/nitrogen (296 K, 1.5 atm) sample for more precise frequency axis calibration as methane absorption peaks are much more spectrally resolved than polypropylene. The measured and reference spectra of methane are shown in Figure A1 (Appendix A). The reference spectra were obtained by simulating methane absorption from the HITRAN spectral database [68] at a sampling interval of 0.328 cm^−1^. By comparing the measured and reference spectra, the frequency axis was calibrated. In the spectral domain, the uncertainty is caused by the spectral separation of individual comb lines, which ranges from 50 to 950 MHz. Therefore, the spectral uncertainty is at most 0.032 cm^−1^ (950 MHz). In comparison, the frequency stability on the measurement timescale is very high (<3 × 10^−5^ cm^−1^ or <1 MHz) and, therefore, it contributes negligibly to the frequency uncertainty.

### 2.3. Experimental Setup

**Figure 2:**
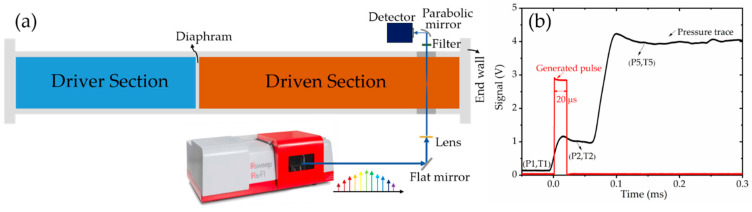
(**a**) Experimental setup consisting of the shock tube and the dual-comb spectrometer. (**b**) A typical pressure signal recorded during the experiment, and the voltage pulse used for synchronization.

The experimental setup used for dual-comb spectroscopy is shown in Figure 2a, where the laser beam passes radially through a shock tube. The shock tube used here is made from stainless steel with an inner diameter of 14.2 cm (optical path length), consisting of two sections, a driver section with an adjustable length up to 9 m and a driven section with a fixed length of 9 m. The shock tube has been described in detail previously [69]. When preparing the shock tube experiment, firstly, the driven section is filled up to an initial pressure of P1 with a target gas sample at an initial temperature of T1. Next, the driver section is pressurized with helium gas until the polycarbonate diaphragm, which separates the two sections, ruptures. A shock wave is initiated which travels at supersonic speeds in the driven section and raises the pressure and temperature of the test gas to P2 and T2, respectively. When this incident shock wave hits the end wall of the shock tube, it gets reflected and causes the second increase of pressure and temperature to P5 and T5, respectively. A typical pressure trace (black solid line) is shown in Figure 2b. The pressure signal is used to generate a voltage pulse, shown as a solid red line in Figure 2b, which triggers the spectrometer and thus synchronizes the shock tube system and the dual-comb spectrometer. The time available for measurements behind reflected shock wave is usually of the order a few milliseconds, after which an expansion wave arrives from the driver section and cools down the test gas. The diaphragm thickness and initial pressure (P1) are varied to achieve the desired temperature and pressure behind the reflected shock wave. In this work, we performed measurements over T ~ 1000–1400 K and P ~ 7 bar.

The sample beam is directed through the shock tube *via* two ZnSe windows mounted at 2 cm from the shock tube end wall. The beam is focused in the center of shock tube using a ZnSe lens to minimize beam-steering effects caused by density gradients inside the shock tube. A bandpass filter (Northumbria Optical Coatings, Ltd) is placed downstream of the shock tube to minimize thermal emission reaching the infrared detector. The transmitted laser is focused by a parabolic mirror onto the detector. The signal captured by the detector is recorded by a high-speed digitizer in the IRis-F1 system. Pure argon shock experiments were carried out to verify minimal perturbance by beam-steering and thermal emission. The IRis-F1 system is triggered with a 3 V, 20 μs pulse produced by a pulse generator. The pulse is initiated by the signal from a Kistler pressure transducer which measures pressure rise by the shock wave; both signals are shown in Figure 2b.

### 2.4. Multi-Species Absorption

The absorbance (*α_ij_ (ν)*) of transition j for a target molecule *i* is obtained using the Beer–Lambert law, Equation (1), by taking the natural log of the ratio of multi-heterodyne signal from the empty beam path (*I_0_*) with that from the sample beam path (*I_t_*).

(1) $$\alpha_{ij}\left(\nu \right)=-ln\left(\frac{I_{t}}{I_{0}}\right)=P\cdot X_{i}\cdot L\cdot S_{ij}\left(T\right)\cdot \phi_{ij}\left(\nu \right),$$

where *P* and *L* are the total pressure and the absorption length, respectively; *X_i_* is the mole fraction of molecule *i* while *S_ij_ (T)* and *ϕ_ij_ (ν)* are line strength and line shape function, respectively; *ν* is a vector containing all wavenumbers contributing to absorbance *α*. Here, *α* is the absolute absorbance obtained from the difference spectra from time-resolved measurements and the initial spectra from static measurement, as described in Section 2.2.

When several species are present in the test sample, and assuming each species absorbs light independently in the optically thin limit [70], the entire test spectrum is considered to be a linear combination of reference spectra from individual species [71]. Accordingly, in this work, the test spectrum (*α_mix_*) is described as Equation (2), where the reference spectra are from four individual samples of methane (CH_4_), propyne (CH_3_CCH), propene (CH_2_CHCH_3_) and acetone (CH_3_COCH_3_), respectively, diluted in nitrogen. In Equation (2), *c_1_, c_2_, c_3_, c_4_* give the contribution of the respective species to the total absorbance while *c_0_* represents an offset. As mole fractions of reference samples are *x_1_, x_2_, x_3_,* and *x_4_,* respectively, *c_i_ ∙ x_i_* (*i* = 1, 2, 3, 4) gives the mole fraction of each species in the test sample, with negligible dependence of the line shape function on species mole fraction in our experimental conditions.

(2) $$\alpha_{mix}\left(\nu \right)=c_{0}+c_{1}\cdot \alpha_{CH_{4}}\left(\nu \right)+c_{2}\cdot \alpha_{CH_{3}CCH}\left(\nu \right)+c_{3}\cdot \alpha_{CH_{2}CHCH_{3}}\left(\nu \right)+c_{4}\cdot \alpha_{CH_{3}COCH_{3}}\left(\nu \right),$$

Since high-temperature cross-sections are not available in the literature for these species, we initially performed experiments with a single target species (e.g., CH_4_ diluted in N_2_). The information from these single-species experiments was then used as reference spectra to determine mole fractions in multi-species experiments. A weighted least-squared fitting is performed on the linear absorption system (Equation (2)) to get all coefficients. The uncertainty of absorbance at each laser frequency is taken as a weighting factor in the least-squared algorithm.

**Figure 3:**
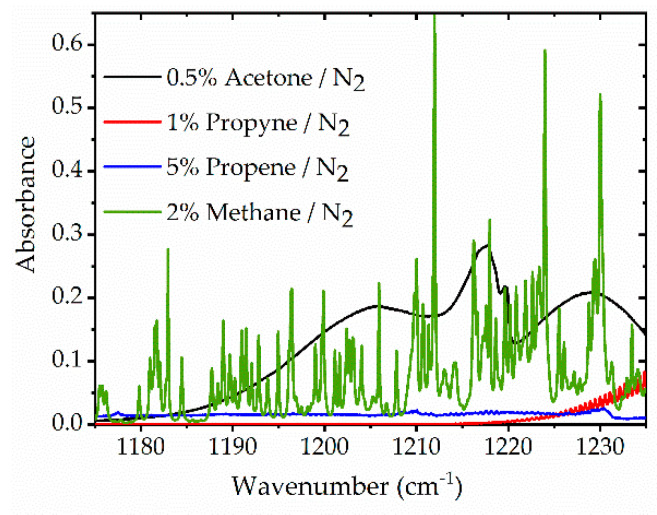
Simulated spectra of acetone, propyne, and propene with a spectral resolution of 0.06 cm^−1^ from the PNNL database [72] at 298 K, 1 atm, and a path-length of 14.2 cm. Methane is simulated with a resolution of 0.01 cm^−1^ at a temperature of 1060 K, a pressure of 7 bar, and a path length of 14.2 cm by using the HITRAN database [68]. Spectra are simulated over the wavenumber range of 1175 to 1235 cm^−1^.

**Figure A2:**
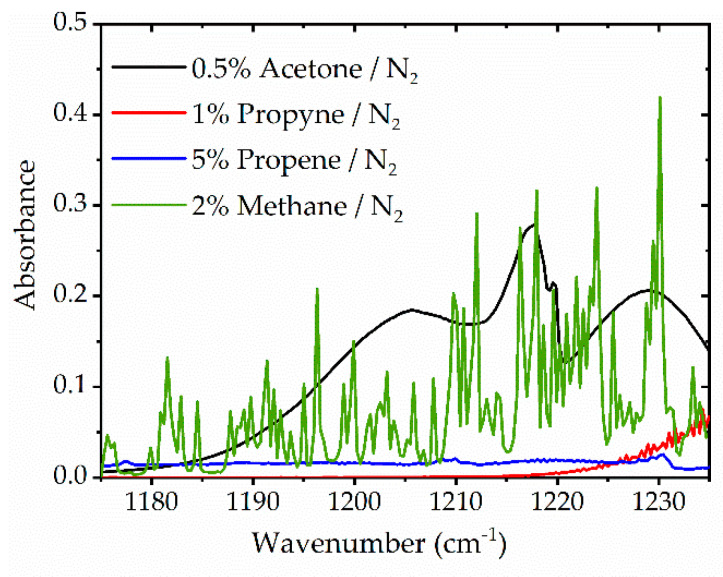
Simulated spectra of acetone, propyne, and propene from the PNNL database [72] at 298 K, 1 atm, and a path-length of 14.2 cm. Methane is simulated at a temperature of 1060 K, a pressure of 7 bar, and a path-length of 14.2 cm by using the HITRAN database [68]. These spectra are simulated at a resolution of 0.328 cm^−1^, the sampling interval of the dual-comb spectrometer.

The simulated spectra of target species are shown in Figure 3 and give a general view of how these four species absorb over the wavelength range of the dual-comb spectrometer. Methane and propyne spectra are more resolved while acetone and propene have relatively broadband spectra. Compared to other species, propene absorption is relatively weak in this wavelength window. The simulated spectra of target species with a spectral resolution of 0.328 cm^−1^, the sampling interval of the spectrometer, are shown in Figure A2 (Appendix A).

## 3. Results and Discussion

### 3.1. Transmission Standard Deviation

**Figure 4:**
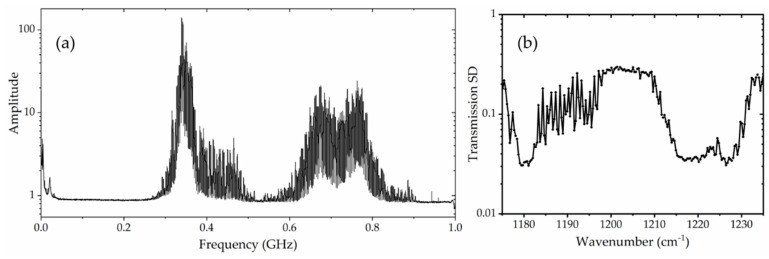
(**a**) A representative RF multi-heterodyne spectrum. The temperatures of the two lasers were set to −3.6 and 21 °C, and the currents were set to 1.015 and 1.116 A, respectively. (**b**) The standard deviation of the transmission signal for an empty beam path of the DCS system at a 4-μs integration time.

A representative RF multi-heterodyne spectrum is shown in Figure 4a. The standard deviation (SD) in the transmission of each spectral line through an empty beam path is shown in Figure 4b. The SD is mainly determined by the intensity profiles of two combs and the detector response. We observe that the SD is quite low in two spectral regions near 1180 and 1220 cm^−1^. The relatively high SD between ~1182–1211 cm^−1^ region is mainly due to the low power of the relevant comb lines, as other noise sources, such as the detector noise floor, have a stronger contribution in weaker spectral elements. The relative power distribution among the laser spectral elements is largely determined by the laser design. The overall uncertainty of species measurements mainly comes from the SD of the transmission signal. Due to the inhomogeneous power distribution among comb lines and the corresponding resulting different relative weights of the spectral elements in the speciation, this noise figure per spectral element is preferred to a single value for the whole system as often used for AM-modulated comb systems [25].

### 3.2. Multi-Species Detection in Non-Reactive Experiments

Multi-species absorption measurements are performed behind reflected shock waves with DCS, as described in Section 2.3. Firstly, individual experiments are carried out for reference gas samples, i.e., 1% acetone/N_2_, 1% propyne/N_2_, 5% propene/N_2_ and 5% methane/N_2_, to measure the reference spectra at a temperature of ~1060 K and pressure of ~7 bar. The temperature is kept low to avoid chemical decomposition of molecules, and relatively high pressure is chosen to collisionally broaden the absorption lines considering the relatively high sampling interval (0.328 cm^−1^) of the DCS system. 

**Figure 5:**
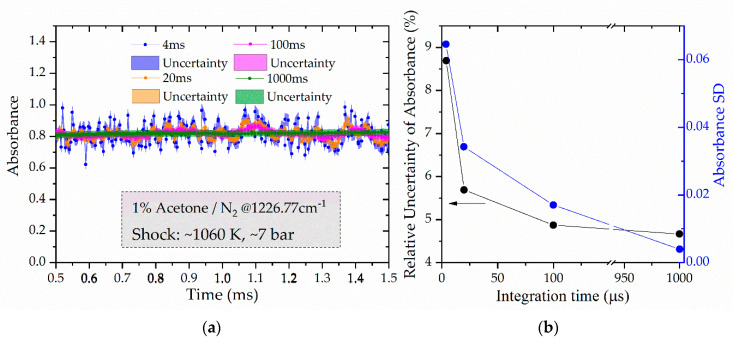
(**a**) Absorbance time-history of acetone at different integration times of 4, 20, and 100 μs at 1226.77 cm^−1^. The shaded areas present the uncertainty of the absorbance measurements. (**b**) The averaged (over a 0.5 to 1.5 ms time window) relative uncertainties of absorbance and the fluctuation (SD) of absorbance as a function of integration time.

Ultimately, measurements are recorded in three dimensions, i.e., absorbance, time, and wavenumber. Species’ absorbance time-history can then be plotted at specific wavenumbers or the absorbance can be analyzed over the entire spectral range (1175–1235 cm^−1^) at certain time windows. Figure 5a shows the absorbance time-history of acetone at 1226.77 cm^−1^ for a shock experiment conducted at 1060 K and 7 atm with a 1% acetone/N_2_ mixture. The data are plotted for various integration times, starting from the fastest possible time resolution of 4 to 100 μs. The shaded area marks the uncertainty of the absorbance measurement. Relatively large fluctuations are seen in this absorbance traces at 1226.77 cm^−1^ due to the relatively high transmission standard deviation at this frequency (see Figure 4b). Increasing the integration time reduces the absorbance fluctuations as would be expected. The relative uncertainty of absorbance, or absorbance SD, is also reduced with increasing integration time, as plotted in Figure 5b. An appropriate integration time needs to be selected based on the trade-off between time resolution required in the measurement and the need for a good SNR.

**Figure 6:**
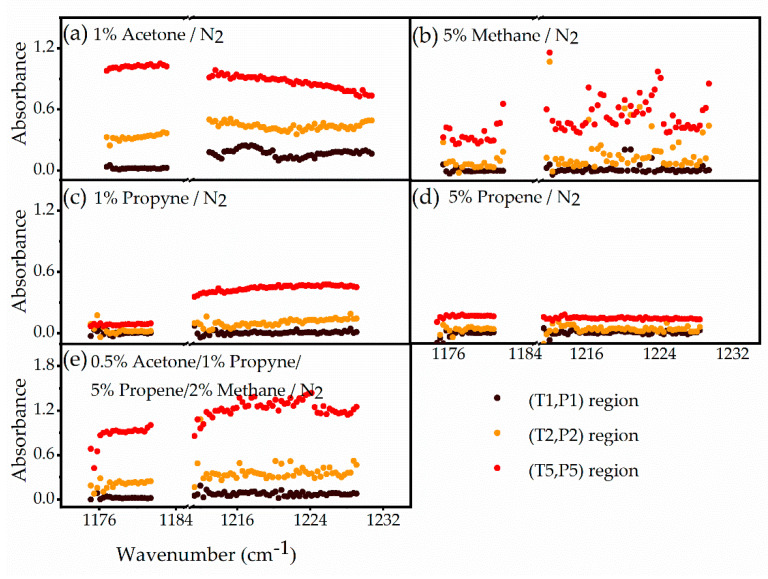
The averaged absorption spectra for four reference samples, (**a**–**d**), and one test gas mixture, (**e**), are shown in the wavenumber ranges of 1175–1182 cm^−1^ and 1212–1230 cm^−1^. Conditions are nominally same for all five experimental spectra: T1 = 296 K, P1 ~ 140 Torr; T2 ~ 660 K, P2 ~ 1.5 bar; T5 ~ 1060 K and P5 ~ 7 bar. The averaging time is 0.2 ms for absorbance results from (T1, P1) region, 65 μs for (T2, P2) region and 1 ms for (T5, P5) region. The fitted spectrum of test gas mixture with linear regression method and the relevant residuals are shown in Figure A3 (Appendix A).

Similar shock tube experiments were carried out for methane, propyne and propene. Measured absorbance values were then averaged in three domains: pre-shock region (T1, P1), post-incident shock region (T2, P2) and post-reflected shock region (T5, P5). The spectra of these four species are plotted in Figure 6a–d as a function of wavenumber in the three T, P regions. All species show increasing absorbance (from region 1 to 2 to 5) of different magnitude due to the combined influence of changes in number density and absorption cross-section with temperature and pressure. As expected, only methane shows resolved spectra (see simulated spectra in Figure 3); however, methane features are also blended due to pressure broadening and relatively large spectral spacing of 0.328 cm^−1^. Spectral data between ~1186–1212 cm^−1^ are not plotted in Figure 6 due to the high uncertainty (or standard deviation) of the spectrometer in this range.

Finally, shock wave experiments were carried out in a test sample comprising of 0.5% acetone/1% propyne/5% propene/2% methane and balance N_2_. These experiments were conducted at conditions similar to those used for measuring the reference spectra, i.e., ~1060 K, and ~7 bar. Utilizing the four reference spectra (Figure 6a–d) and the test mixture spectrum (Figure 6e), the mole fractions of the four species in the test sample are determined based on Equation (2) with weighted least-squared fitting method and considering the relevant uncertainty values. The results are summarized in Table 1. The resulting mole fractions are found to be within a deviation of 10% from the known mixture composition. The availability of wide spectral measurements enabled us to calculate quantitative mole fraction values despite the largely broadband shape of the individual spectra. These results validate the applicability of such a dual-comb spectrometer for time-resolved multi-species measurements at high temperatures and high pressures.

### 3.3. Application to Reactive Experiments

**Figure 7:**
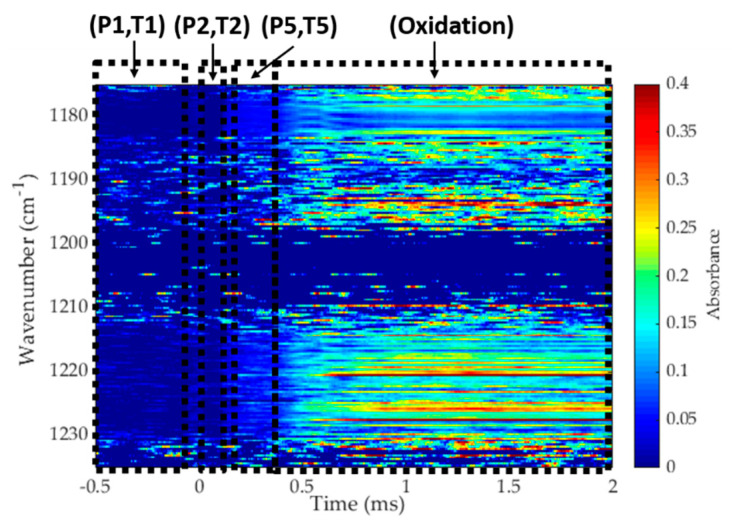
Absorbance spectra measured by the mid-IR DCS during the oxidation of 1% propene/O_2_/argon (ϕ = 1) mixture at T1 = 296 K, P1 = 68 Torr; T2 = 826 K, P2 = 1.06 bar; T5 = 1390 K and P5 = 5.6 bar. Different chemical kinetic phases may be recognized through this 3D plot.

To explore the potential of this dual-comb spectrometer to capture rapid chemical kinetic processes, the spectrometer was utilized to measure absorbance time histories during the oxidation of 1% propene/O_2_/argon (ϕ = 1) mixture at a temperature of 1389 K and pressure of 5.5 atm. Measured absorbance is plotted in Figure 7 as a function of wavenumber and time (where time zero is the arrival of reflected shock wave). The pressure trace (not shown) indicated ignition taking place at about 0.8 ms for this experiment. Several interesting features can be observed in this spectrum. In the pre-shock region (−0.5 to 0 ms), there is very little absorption coming only from propene. After the reflected shock wave, some increased absorbance is observed for propene (e.g., near 1180 cm^−1^) which quickly goes away as propene is consumed during the oxidation process. Beyond 0.4 ms, larger absorbance signals are caused by the reactive process picking up steam and the conversion of propene into smaller intermediate hydrocarbons (e.g., methane, ethylene, acetylene). After ignition (~0.8 ms), absorbance is increased considerably at specific wavenumbers and this increase primarily comes from the spectrally resolved spectra of water vapor in this region. These measured spectra were not converted to mole fraction as this would require the knowledge of high-temperature spectra of propene, intermediate hydrocarbons and products. However, such a measurement demonstrates the power of a broadband spectral source for investigating complex chemical reaction systems. 

## 4. Conclusions

We have demonstrated a quantum cascade laser based dual-comb spectrometer, emitting from 1175 to 1235 cm^−1^ with a spectral sampling of ~0.328 cm^−1^ at a time resolution of 4 μs, for multi-species detection at high temperatures and pressures in a shock tube. We characterized the spectrometer by measuring the absorbance standard deviation and calibrating the frequency axis to methane spectral lines. Non-reactive shock tube experiments were carried out to demonstrate time-resolved simultaneous measurements of four species in a gas mixture at a temperature of 1060 K and pressure of 7 atm. Finally, the spectrometer was utilized in a representative study of propene oxidation to show the potential of this strategy in resolving rapid chemical processes of reacting systems. Future work will focus on improving the absorbance standard deviation and increasing the SNR at low integration times.

## Figures and Tables

**Table 1:** Measured and expected mole fractions of all four species from the test sample.

Species	Mole Fraction (Measured)	Mole Fraction (Expected)	Relative Error (%)
Acetone	0.55%	0.5%	9.1%
Propyne	0.93%	1%	7.0%
Propene	4.70%	5%	6.1%
Methane	2.19%	2%	9.5%

**Figure A3:**
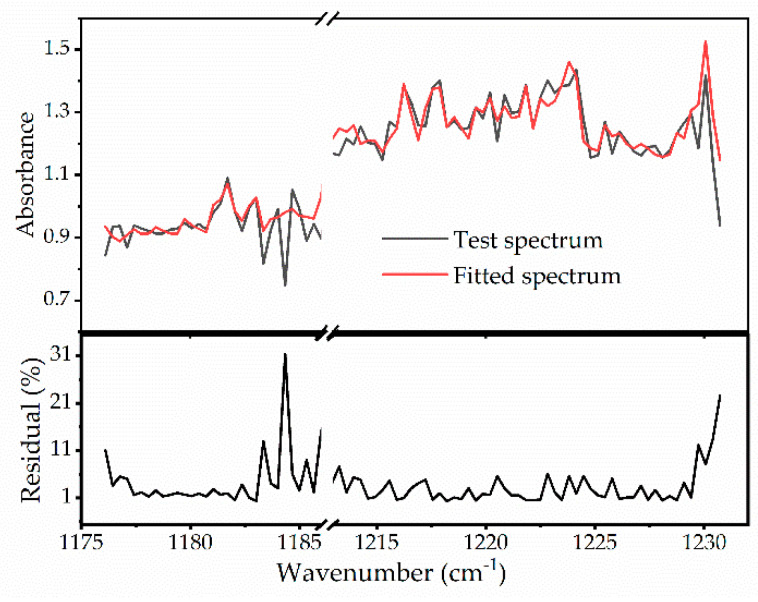
Upper panel: The measured absorbance (black line) of the test sample, 0.5% acetone/1% propyne/5% propene/2% methane with balance N_2_; the fitted absorbance (red line) obtained by fitting test spectrum using four reference spectra with weighted linear regression method. Bottom panel: The residual or the relative discrepancy of fitted absorbance from the measured absorbance. Residuals around 1185 cm^−1^ and 1230 cm^−1^ are high due to the relatively high standard deviation in the relevant transmission lines (shown in Figure 4b).
